# Is prior bariatric surgery associated with poor COVID-19 outcomes? A systematic review and meta-analysis of case-control studies

**DOI:** 10.7189/jogh.13.06012

**Published:** 2023-04-14

**Authors:** Xiang Gao, Pengzhou Li, Song Dai, Guohui Wang, Weizheng Li, Zhi Song, Liyong Zhu, Shaihong Zhu

**Affiliations:** Department of General Surgery, Third Xiangya Hospital, Central South University, Changsha, China

## Abstract

**Background:**

Obesity is an independent risk factor for severe coronavirus disease 2019 (COVID-19), but there is little evidence on whether prior bariatric surgery benefits the outcomes of patients with COVID-19. We aimed to summarize this relationship by conducting a systematic review and meta-analysis of current case-control studies.

**Methods:**

We searched several electronic databases for case-control studies conducted between January 2020 and March 2022. We compared the rates of mortality, mechanical ventilation, intensive care unit (ICU) admission, dialysis, hospitalization, and length of hospital stay between COVID-19 patients with and without a history of bariatric surgery.

**Results:**

We included six studies with 137 903 patients; 5270 (3.8%) had prior bariatric surgery, while 132 633 (96.2%) did not. COVID-19 patients with a history of bariatric surgery had significantly lower mortality (odds ratio (OR) = 0.42; 95% confidence interval (CI) = 0.23-0.74), ICU admission (OR = 0.48; 95% CI = 0.36-0.65), and mechanical ventilation rates than those with a history of non-bariatric surgery (OR = 0.51; 95% CI = 0.35-0.75).

**Conclusions:**

Prior bariatric surgery was associated with a reduced risk of mortality and reduced severity of COVID-19 in patients with obesity compared to those with no prior bariatric surgery. Further large-sample prospective studies are needed to support these results.

**Registration:**

CRD42022323745.

The coronavirus disease 2019 (COVID-19) pandemic, caused by severe acute respiratory syndrome coronavirus 2 (SARS-CoV-2), has become a global public health crisis [1]. A recent study suggested that 18.2 million COVID-19-related deaths occurred between 1 January 2020 and 31 December 2021 (as measured by excess mortality), which is thrice the number of officially recorded deaths during that period (n = 5.94 million) [2]. Numerous studies have shown that obesity and many obesity-related comorbidities, such as type 2 diabetes (T2D), heart disease, hypertension, kidney disease, dyslipidaemia, and respiratory dysfunction, have been associated with an increased risk of poor outcomes and death due to COVID-19 [3,4].

Bariatric surgery is increasingly being performed in patients with morbid obesity worldwide. However, there is little evidence of COVID-19 outcomes in patients who went through bariatric surgery. This procedure can lead to successful long-term weight loss [5,6], improved metabolism (including T2D remission [7] and better control of blood pressure and lipids [8,9]), improved sleep apnoea syndrome [10], and reduced systemic chronic inflammation [11]. Consequently, it is plausible that prior bariatric surgery may have a protective effect in obese patients infected with SARS-CoV-2. However, bariatric surgery can lead to vitamin deficiencies and malnutrition [12-14], and studies have reported that vitamin D deficiency increases the risk of COVID-19 infection and death [15] Furthermore, the hypercatabolic and immunosuppression states following bariatric surgery are important risk factors for poor clinical outcomes of COVID-19 [16,17]. Guidelines have been published for prioritizing patients who should undergo bariatric surgery based on diseases that are most likely to be ameliorated postoperatively during the COVID-19 pandemic [18]. To our knowledge, no large-scale study has been conducted on whether bariatric surgery impacts the severity of COVID-19. We aimed to systematically review and meta-analyse all existing published clinical data to assess the effect of prior bariatric surgery on COVID-19 in patients with obesity.

## METHODS

We conducted this systematic review and meta-analysis following the Preferred Reporting Items for Systematic Reviews and Meta-analyses (PRISMA) guidelines [19] and registered it in the International Prospective Register of Systematic Reviews (CRD42022323745). Two authors (XG, PL) independently conducted all steps of the literature search, study selection, data extraction, and quality assessment, resolving disagreements with the third author (GW).

### Search strategy

We searched EMBASE, PubMed, the Cochrane Library, and Web of Science from January 2020 through March 2022 using appropriate medical subject heading (MeSH) terms and free text in all fields, without limitations for study design; some of the search terms were: “bariatric surgery”, “gastric bypass”, “Roux-en-Y”, “metabolic surgery”, “COVID-19”, “SARS-CoV-2”, “2019 Novel Coronavirus Disease”, “Severe Acute Respiratory Syndrome Coronavirus 2 Infection” (the detailed search strategy is described in supplement 1 of the **Online Supplementary Document**). We performed the last search in April 2022.

### Inclusion criteria

We included only original English-language case-control studies that contained clinical outcome data of patients with COVID-19 after bariatric surgery. Study participants were adults older than 18 years. We included only studies published from January 2020.

### Exclusion criteria

We excluded abstracts, conference articles, opinion pieces, editorial letters, case studies, reviews, and meta-analyses from the final analysis. No case-control studies were excluded. We also excluded studies with missing data related to the primary and secondary study outcomes.

### Outcomes assessed

The primary outcome was mortality due to COVID-19 in patients with obesity and prior bariatric surgery. Secondary outcomes included the following: admission to the intensive care unit (ICU); dialysis during hospitalization; requirement for mechanical ventilation; hospitalization; and length of hospital stay.

### Data extraction

Two researchers independently screened the titles and abstracts of the retrieved studies for eligibility, discussing discrepancies with the research team members until consensus was reached. Then, both researchers screened the full texts of all potentially relevant against eligibility criteria, while a third author independently assessed the studies for eligibility. Two authors independently extracted the following data from the included studies: author(s), year of publication, region and country, sample size, mortality, hospitalization, ICU admission, dialysis and mechanical ventilation rates, length of hospital stay, mean age, standard deviation of age, mean body mass index (BMI), and COVID-19 diagnosis.

### Quality assessment-risk of bias

We used the Newcastle–Ottawa Scale (NOS) [20] to assess the quality of the included case-control studies. Two researchers independently assessed them for study selection, comparability, and outcomes, scoring eight items with three subscales, with a maximum total score of 9. We considered a study scoring ≤3 to be of poor quality, 4-6 to be of fair quality, and ≥7 to be of good quality.

### Statistical analysis

We performed the statistical analysis using RevMan 5.4, setting the statistical significance at *P* < 0.05. We calculated odds ratio (OR) and 95% confidence intervals (CI) for categorical clinical data and mean differences for continuous clinical data. We quantitatively pooled the outcome measures, using a random-effects model or fixed-effect model when possible, depending on heterogeneity among the studies. We assessed heterogeneity with the *I*^2^ statistic, with ≥75% indicating high heterogeneity. We did not assess publication bias since we included less than 10 studies in the meta-analysis. Based on study quality assessments, we performed a sensitivity analysis using the “leave-one-out” approach.

## RESULTS

### Study selection

**Figure 1:**
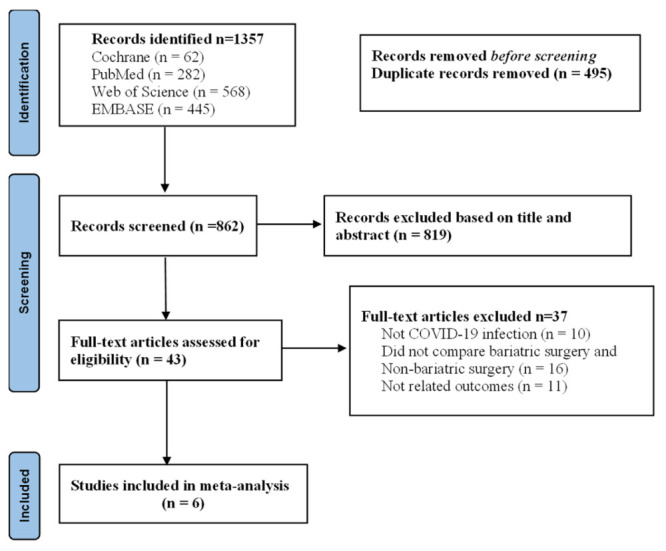
Meta-analysis flow diagram outlining the search strategy and results of the search.

We comprehensively and systematically searched PubMed, Web of Science, Embase, and the Cochrane Library from January 2020 to March 2022 and initially identified 1357 articles. After the exclusion process, we included six studies [21-26] in this meta-analysis (**Figure 1**).

### Characteristics of the included studies

The studies included 137 903 patients with obesity and COVID-19; 5270 (3.8%) had previously undergone bariatric surgery before COVID-19 infection while 132633 (96.2%) did not. There were 4030 (76.5%) females in the bariatric surgery group and 69 723 (52.6%) in the non-bariatric surgery group. The mean ages of the patients in the included studies ranged from 46.1 (standard deviation (SD) = 12.7) to 51.7 (SD = 12.6) for the bariatric surgery group and from 45.1 (SD = 10.1) to 59.8 (SD = 12.4) for the non-bariatric surgery group. Four studies were conducted in the United States, one in France, and one in Iran. In three studies, bariatric surgery patients were propensity matched to non-bariatric surgery patients. More detailed information on the baseline characteristics is described in **Table 1**.

**Table 1:** Characteristics of all the included studies in this meta-analysis

					Patients (n)		Female		Age in years, mean (SD)		Mean BMI in kg/m^2^, mean (SD)		
**Study, year**	**Country**	**Type of study**	**Type of surgery**	**Race**	**BS**	**Non-BS**	**BS**	**Non-BS**	**BS**	**Non-BS**	**BS**	**Non-BS**	**NOS**
Aminian et al, 2021 [21]	United States	Retrospective, matched cohort	RYGB, SG	White, Black, other	33	330	26	254	46.1 (12.7)	48.8 (14.7)	37.2 (7.1)	42.3 (7.0)	9
Hadi et al, 2022 [22]	United States	Retrospective, matched cohort	RYGB, SG, GB	African American, Caucasian, Hispanic or Latino	1940	1940	1597	1603	48.13 (11.88)	48.62 (12.43)	NA (BMI>35, n = 1292)	NA (BMI>35, n = 1292)	8
Iannelli et al, 2020 [23]	French	Retrospective, population-based, multi-institutional, cohort	RYGB, GB, SG	NA	541	7745	414	3576	49.8 (12.0)	59.8 ± 12.4	NA (BMI>30, n = 541)	NA (BMI>30, n = 6667)	9
Jenkins et al, 2021 [24]	United States	Retrospective, matched cohort	RYGB, GB, SG	Spanish/Hispanic, Black, White	124	496	86	342	51.7 (12.6)	52.1 (12.9)	36.1 (8.3)	41.4 (6.5)	8
Moradpour et al, 2021 [25]	Iran	Retrospective matched cohort	RYGB, SG		25	30	19	23	45.3 (11.3)	45.1 (10.1)	29.65 (6.2)	45.08 (5.8 =	7
Purdy et al, 2021 [26]	United States	Retrospective cohort	NA	White, Black, Hispanic Asian, other or unknown	2607	12 2092	1888	63 925	NA (18-64 y (n = 1888), >65 y (n = 719))	NA (18-64 y (n = 7813), >65 y (n = 43 960))	NA	NA	9

SD – standard deviation, BMI – body mass index, NOS – Newcastle-Ottawa Scale, BS – bariatric surgery, non-BS – non-bariatric surgery, RYGB – Roux-en-Y gastric bypass, GB – gastric banding, SG – sleeve gastrectomy, y – years, NA – not applicable

### Mortality

**Figure 2:**
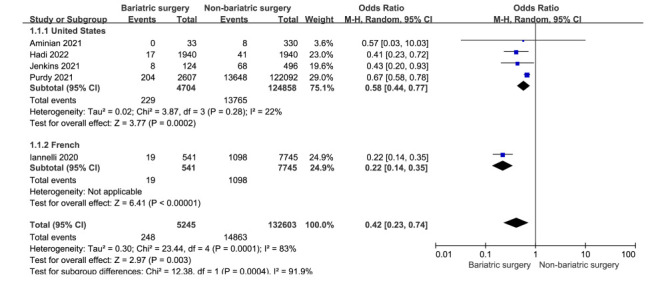
A meta-analysis of mortality rates in patients with COVID-19 and prior bariatric surgery or non-bariatric surgery.

We included five studies in the mortality analysis; the random-effects model showed a lower mortality rate in patients with COVID-19 with prior bariatric surgery than in patients without prior bariatric surgery (OR = 0.42; 95% CI = 0.23 to 0.74, *I*^2^ = 83%). The results of the subgroup analysis based on the study countries are shown in **Figure 2**. In the United States, the bariatric surgery group had a lower mortality rate than the non-bariatric surgery group (OR = 0.58; 95% CI = 0.44 to 0.77). There was low heterogeneity (*I*^2^ = 22%, *P* = 0.28).

### ICU admission

**Figure 3:**
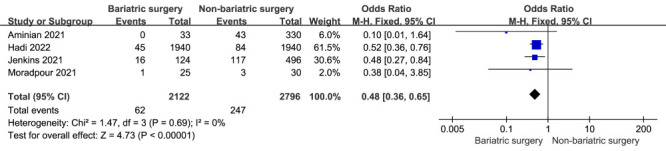
A meta-analysis of ICU admission rates in patients with COVID-19 and prior bariatric surgery or non-bariatric surgery.

Four studies reported ICU admission rates between patients with obesity and COVID-19 with prior bariatric surgery and non-bariatric surgery. Patients with obesity and COVID-19 with prior bariatric surgery were significantly less likely to be admitted to the ICU (OR = 0.48; 95% CI = 0.36 to 0.65; *P* < 0.00001) (**Figure 3**) [21,22,24,25]. There was no evidence of heterogeneity (*I*^2^ = 0.0%, *P* = 0.69).

### Dialysis

**Figure 4:**
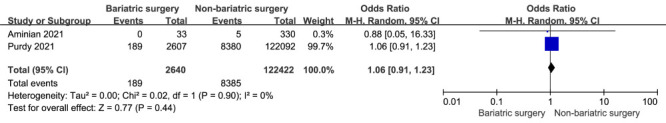
A meta-analysis of dialysis rates in patients with COVID-19 and prior bariatric surgery or non-bariatric surgery.

Two studies reported on dialysis rates [21,26]. Compared to patients with non-bariatric surgery, patients with obesity, COVID-19, and prior bariatric surgery did not show significantly different hospitalization rates (OR = 1.06; 95% = CI = 0.91 to 1.23; *P* = 0.44; *I*^2^ = 0%) (**Figure 4**).

### Mechanical ventilation

**Figure 5:**
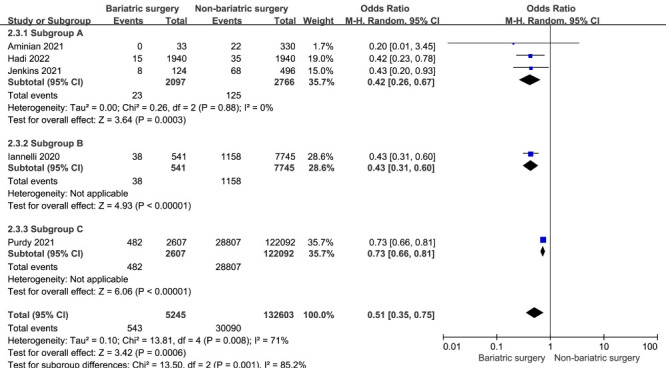
A meta-analysis of mechanical ventilation rates in patients with COVID-19 and prior bariatric surgery or non-bariatric surgery.

Five studies reported on mechanical ventilation rates [21-24,26]. Compared to non-bariatric surgery, prior bariatric surgery was significantly less likely to result in mechanical ventilation in patients with obesity and COVID-19 (OR = 0.51; 95% CI = 0.35 to 0.75; *P* = 0.0006; *I*^2^ = 71%) (**Figure 5**).

### Hospitalization

**Figure 6:**
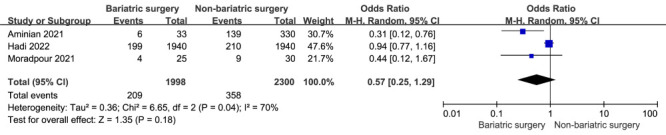
A meta-analysis of hospitalization rates in patients with COVID-19 and prior bariatric surgery or non-bariatric surgery.

There was no significant difference in hospitalization rates between patients with obesity and COVID-19 with prior bariatric surgery and non-bariatric surgery (three studies [21,22,25], 4328 participants, OR = 0.57; 95% CI = 0.25 to 1.29; *P* = 0.18). There was evidence of heterogeneity (*I*^2^ = 70%, *P* = 0.04) (**Figure 6**).

### Length of hospital stay

**Figure 7:**
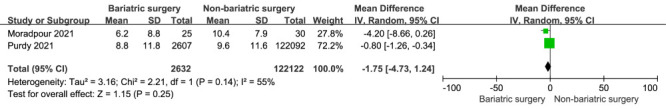
A meta-analysis of the length of hospital stays in patients with COVID-19 and prior bariatric surgery or non-bariatric surgery.

There was no significant difference in the length of hospital stay between patients with obesity and COVID-19 with prior bariatric surgery and non-bariatric surgery (two studies [25,26], 124 754 participants, mean difference (MD)  = -1.75; 95% CI = −4.73 to 1.24, *P* = 0.25). There was evidence of heterogeneity (*I*^2^ = 55%, *P* = 0.14) (**Figure 7**).

### Publication bias and sensitivity analyses

The pooled results of mortality, mechanical ventilation, and hospitalization in our meta-analysis showed high heterogeneity. The most popular approach to assess publication bias is the funnel plot. However, because the meta-analysis included fewer than 10 studies, we could not assess the outcomes for potential publication bias. Under these circumstances, we performed the sensitivity analysis of the outcomes to identify potential sources of bias, assess the robustness of the outcomes, and identify the effect of any one study on the pooled effect size and between-study heterogeneity by excluding each study one by one for each outcome. We found that the mortality, ICU admission, and mechanical ventilation results were robust, with only small changes in the ORs when individual studies were excluded. When excluding the study by Hadi et al. [22], we found significant differences in the results of the hospitalization outcomes (OR = 0.5; 95% CI 0.35 to 0.75, *P* = 0.005, *I*^2^ = 0) (**Table 2****)**.

**Table 2:** Sensitivity analysis of the outcomes to assess robustness

	Study excluded	OR (95% CI)	*P*-value	*I*^2^ statistic (%)
**Mortality**	All studies included (before sensitivity analysis)	0.42 (0.23 to 0.74)	0.003	83
	Aminian et al, 2021 [21]	0.41 (0.22 to 0.75)	0.004	87
	Hadi et al, 2022 [22]	0.42 (0.19 to 0.89)	0.02	86
	Iannelli et al, 2020 [23]	0.58 (0.44 to 0.77)	0.0002	22
	Jenkins et al, 2021 [24]	0.41 (0.20 to 0.83)	0.01	87
	Purdy et al, 2021 [26]	0.32 (0.21 to 0.48)	<0.00001	27
**ICU admission**	All studies included (before sensitivity analysis)	0.48 (0.36 to 0.65)	<0.00001	0
	Aminian et al, 2021 [21]	0.51 (0.37 to 0.69)	<0.0001	0
	Hadi et al, 2022 [22]	0.42 (0.24 to 0.71)	0.001	0
	Jenkins et al, 2021 [24]	0.48 (0.34 to 0.69)	<0.0001	0
	Moradpour et al, 2021 [25]	0.48 (0.36 to 0.66)	<0.00001	0
**Mechanical ventilation**	All studies included (before sensitivity analysis)	0.51 (0.35 to 0.75)	0.0006	71
	Aminian et al, 2021 [21]	0.52 (0.35 to 0.77)	0.001	77
	Hadi et al, 2022 [22]	0.54 (0.35 to 0.83)	0.005	73
	Iannelli et al, 2020 [23]	0.57 (0.38 to 0.85)	0.006	46
	Jenkins et al, 2021 [24]	0.53 (0.34 to 0.81)	0.003	76
	Purdy et al, 2021 [26]	0.43 (0.32 to 0.56)	<0.00001	0
**Hospitalization**	All studies included (before sensitivity analysis)	0.57 (0.25 to 1.29)	0.18	70
	Aminian et al, 2021 [21]	0.87 (0.55 to 1.37)	0.55	17
	Hadi et al, 2022 [22]	0.51 (0.35 to 0.75)	0.005	0
	Moradpour et al, 2021 [25]	0.59 (0.20 to 1.75)	0.34	82

OR – odds ratio, CI – confidence interval

## DISCUSSION

This meta-analysis provides evidence that prior bariatric surgery reduces the risks of mortality, mechanical ventilation, and ICU admission in patients with COVID-19. There were no significant differences between the bariatric surgery group and the non-bariatric surgery group in the rate dialysis, rate of hospitalization, or length of hospital stay among COVID-19 patients. The COVID-19 pandemic has become a global public health crisis, and obesity has been identified as an independent risk factor for severe COVID-19 and a poor prognosis [27,28]. Consequently, we suggest that patients with obesity, especially those with its severe form, take effective measures to prevent COVID-19 infection and control their weight or consider undergoing bariatric surgery.

In our meta-analysis, we found that prior bariatric surgery was significantly associated with mortality due to COVID-19, with a pooled OR of 0.42 (95% CI = 0.23 to 0.74, *P* = 0.003) and high heterogeneity (*I*^2^ = 83%). To discover the source of heterogeneity and whether it impacted our results, we performed subgroup analysis by study region, stratifying patients in the United States and France. The results showed that the heterogeneity was significantly reduced to 22%, and the combined OR value slightly changed; the results were all statistically significant. We speculate that the difference in the mortality rate of COVID-19 was due to differences in COVID-19 severity, national medical assistance guarantees, public health measure effectiveness, and COVID-19 vaccination coverage among different countries and regions [29,30]. Furthermore, our sensitivity analysis also showed that the results were robust, which can explain why patients with COVID-19 with a history of bariatric surgery had a lower mortality rate.

Additionally, prior bariatric surgery was associated with severe COVID-19, including the risks of ICU admission and mechanical ventilation, with pooled ORs of 0.48 (95% CI = 0.36 to 0.65, *I*^2^ = 0%) and 0.51 (95% CI = 0.35 to 0.75, *I*^2^ = 71%), respectively. Regarding mechanical ventilation, although there was moderate heterogeneity among the included studies, we could not analyse the possible sources of the differences by subgroup analysis because they did not consider the severity of COVID-19, comorbidities, and chronic pulmonary disease. Hence, after the stepwise exclusion of studies in the sensitivity analysis, the pooled OR values for mechanical ventilation fluctuated between 0.43 and 0.57, while the results remained statistically significant (*P* < 0.05), indicating reliability.

Our results showed no significant difference in the haemodialysis rate, hospitalization rate, or length of hospital stay between the two groups. Because of the high heterogeneity among studies that reported hospitalization rates, we found that, when the study by Hadi et al. [22] was excluded in the sensitivity analysis, the results changed significantly; the OR value decreased from 0.57 to 0.51, with statistical significance, but we still included this study in the analysis. Since relevant randomized controlled studies have not yet been published, we cannot determine whether there are differences in hospitalization rates in COVID-19 patients after bariatric surgery. It has been nearly three years since the COVID-19 pandemic started, and we speculate that the included studies did not consider potential influencing factors, such as SARS-CoV-2 mutations, personal health protection, and vaccination. Currently, numerous studies have revealed that vaccination against COVID-19 can greatly reduce the severity of COVID-19 [31].

The matching factors for the included studies are described in **Table 3**. it is well known that age, gender, and ethnicity factors have an important impact on the prognosis of many diseases. The studies included in this meta-analysis all consider the influence of age and sex, while Hadi et al. [22] and Iannelli et al. [23] also match BMI, while Aminian et al. [21], Hadi et al. [22], and Purdy et al. [26] also matched race. The matching of these potential confounding factors can effectively reduce the influence of bias and improve the reliability of the results. Moreover, an important issue to consider is the difference in comorbidities, but it is relatively difficult to match co-morbidities, such as diabetes, hypertension, cardiovascular disease, chronic obstructive pulmonary disease, chronic kidney disease and other comorbidities. The comorbidities of all patients included in the study are listed in **Table 3**. All studies have considered the complications of hypertension and diabetes in obesity, because hypertension and diabetes are the most common comorbidities in obesity which also significantly impact the prognosis of COVID-19. Hypertension and diabetes are especially common among patients, as bariatric surgery can significantly improve obesity-related complications [32], and these diseases are also considered risk factors for severe COVID-19 [33]. Therefore, it could also explain that a history of bariatric surgery may have a protective effect in patients infected with COVID-19.

**Table 3:** Matching factors and co-morbid conditions of included studies in this meta-analysis

	Matching factors	Co-morbidities	Exclusions
Aminian et al, 2021 [21]	Age, sex, race, ethnicity, location (Ohio vs Florida), smoking, COPD, asthma, cancer	Hypertension, diabetes, coronary artery disease, heart failure	NA
Hadi et al, 2022 [22]	Age, race, sex, BMI, diabetes, hypertension, chronic lung diseases, nicotine dependence, heart failure, ischaemic heart disease	Hypertension, chronic lung diseases, heart failure, ischemic heart disease	NA
Iannelli et al, 2020 [23]	Age, sex, BMI	Hypertension, diabetes, COPD, cardiac failure, cancer,	NA
Jenkins et al, 2021 [24]	Age, sex	Hypertension, diabetes, hyperlipidaemia, history of MI, history of stroke	NA
Moradpour et al, 2021 [25]	Age, sex	Hypertension, diabetes, obstructive sleep apnoea, hyperlipidaemia	Severe hepatic, renal impairments, cardiovascular, cerebrovascular diseases, asthma
Purdy et al, 2021 [26]	Age, sex, race/ethnicity	Hypertension, diabetes, chronic pulmonary disease, congestive heart failure, renal disease	NA

COPD – chronic obstructive pulmonary disease, BMI – body mass index, MI – myocardial infarction, NA – not applicable

To our knowledge, this is the first comprehensive meta-analysis of matched case-control studies to directly compare patients with obesity with COVID-19 who underwent bariatric surgery or non-bariatric surgery. Therefore, there are no relevant meta-analyses with which we can compare our results. Lifestyle modification after bariatric surgery is also a potentially important factor. The impact on lifestyle mainly includes a reduction in calorie intake and physical activity [34]. Studies have shown that physical activity alone has a negligible effect on body weight, not combined with calorie restriction [35,36]. Bariatric surgery can significantly affect weight loss and improve metabolic syndrome, so the mechanism needs to be further explored. From our results, we can conclude that bariatric surgery has had a protective effect on obesity during the COVID-19 pandemic. Presently, the different socioeconomic conditions in different countries and the popularity of bariatric metabolic surgery will affect patient acceptance. A cost-effectiveness study [37] showed that undergoing bariatric surgery reduced COVID-19-related morbidity and mortality, as well as obesity-related comorbidities, which indirectly supports our results, indicating the protective effect of bariatric surgery on COVID-19.

A retrospective matched cohort study from the Cleveland Centre for Clinical Research by Aminian et al. [21] matched for age, sex, race, ethnicity, location smoking status, and history of chronic obstructive pulmonary disease (COPD), asthma, or cancer, demonstrating that a history of metabolic surgery was associated with a lower severity of COVID-19 infection in patients with obesity, as manifested by a lower risk of hospitalization and ICU admission. However, the study did not account for drug treatment, vaccination, and post-operative complications. We generally believe that the possible causes of better COVID-19 outcomes are metabolic improvement and the remission of comorbidities. The bariatric group in the study by Hadi et al. [22] had an older age and higher rates of diseases (including ischaemic heart disease and diabetes mellitus) than the non-bariatric surgery group. Therefore, it can be inferred that the protective effect of bariatric surgery on COVID-19 may not be related to systemic metabolic improvement and the improvement in other comorbidities, but rather to susceptibility to SARS-CoV-2 caused by the bariatric surgery itself. Studies have shown that angiotensin-converting enzyme 2 (ACE2), the receptor for SARS-CoV-2, is more highly expressed in adipose tissue than in lung tissue and may be associated with the progression of severe COVID-19 in obese patients [38]. Kristem et al. [39] analysed GSE59034 microarray data in the Gene Expression Omnibus database and found that the proportion of ACE2 receptors was significantly lower in subcutaneous white adipose tissue in obese individuals after Roux-en-Y gastric bypass surgery than in non-obese matched controls. Additionally, a randomized controlled trial reported that weight loss induced a decline in subcutaneous adipose ACE-2 expression [40]. This finding is supported by the results of a single-center cross-sectional study [41] that showed that patients who had undergone bariatric surgery had lower rates of COVID-19.

The strength of this meta-analysis is that we exclusively included case-control studies. Furthermore, by matching for age, sex, ethnicity, and comorbidities with a control group, we managed to exclude (to a certain extent) confounding factors that could potentially lead to uncertainty in the results. It quantitatively summarized available evidence of the protective effect of prior bariatric surgery on COVID-19. However, there are several limitations to our meta-analysis. Due to few relevant studies published thus far, we only included six valid studies, all of which were retrospective cohort studies; there were no randomized controlled studies, as larger randomized controlled trials are unlikely to be carried out due to significant financial cost and the enormous difficulties posed by COVID-19 control policies in various regions. For retrospective studies, the existence of confounding factors is an unavoidable problem; we did not consider the influence of lifestyle in this meta-analysis. The change of lifestyle after bariatric metabolic surgery also has a certain positive effect on the improvement of systemic metabolism, which will also help reduce the incidence of adverse prognosis in patients with COVID-19. Finally, most of the included articles were from the United States, which could have possibly led to publication bias. Thus, we performed sensitivity analysis and subgroup analyses; the results did not change significantly, which further confirms the accuracy and robustness of our results.

## CONCLUSIONS

This meta-analysis indicates that infection with COVID-19 in patients with obesity with prior bariatric surgery reduced the risk of poor clinical outcomes, including mortality, mechanical ventilation, and ICU admission, which may highlight the need for better disease prevention and weight control in patients with obesity. Stronger evidence from larger samples and prospective studies is needed to further support our results. In the future, we will determine which type of bariatric surgery is most effective and how much time is needed to achieve the beneficial effects of obesity surgery on COVID-19.

## Additional material


Online Supplementary Document
