# Supplementary material for: Is prior bariatric surgery associated with poor COVID-19 outcomes? A systematic review and meta-analysis of case-control studies
Source: J Glob Health. 2023 Apr 14;13:06012. doi: 10.7189/jogh.13.06012 (PMC10104427; doi:10.7189/jogh.13.06012)
Supplement: Online Supplementary Document [file jogh-13-06012-s001.pdf]

PUBMED

#1 "COVID-19"[Mesh] OR “COVID 19” OR “COVID19” OR “SARS-CoV-2” OR “SARS CoV 2” OR “2019 Novel Coronavirus Disease” OR “2019 Novel Coronavirus Infection” OR “2019-nCoV Disease” OR “2019 nCoV Disease” OR “2019-nCoV Diseases” OR “2019-nCoV” OR “Coronavirus Disease 2019” OR “Coronavirus Disease-19” OR “Coronavirus Disease 19” OR “Severe Acute Respiratory Syndrome Coronavirus 2 Infection” OR “SARS Coronavirus 2 Infection” OR “2019-nCoV Infection” OR “2019 nCoV Infection” OR “2019-nCoV Infections”

#2 "Bariatric Surgery"[Mesh] OR “bariatric surgery” OR gastroplast∗ OR “gastric bypass” OR “Roux-en-Y” OR “gastric band” OR “biliopancreatic diversion” OR gastrectom∗ OR “duodenal switch” OR “gastrointestinal diversion” OR gastroenterostom∗ OR “jejunoileal bypass” OR “obesity surgery” OR “weight loss surgery” OR “weight-loss surgery” OR “bariatric procedure” OR “sleeve surgery” OR “metabolic surgery”

#3= #1 AND #2

WOS

#1 ((((((((((((((((TS=(Bariatric Surgery)) OR TS=(gastroplast*)) OR TS=(gastric bypass)) OR TS=(Roux-en-Y)) OR TS=(gastric band)) OR TS=(biliopancreatic diversion)) OR TS=(gastrectom*)) OR TS=(duodenal switch)) OR TS=(gastrointestinal diversion)) OR TS=(gastroenterostom*)) OR TS=(jejunoileal bypass)) OR TS=(obesity surgery)) OR TS=(weight loss surgery)) OR TS=(weight-loss surgery)) OR TS=(bariatric procedure)) OR TS=(sleeve surgery)) OR TS=(metabolic surgery)

#2((((((((((((((((((TS=(COVID-19)) OR TS=(COVID 19)) OR TS=(COVID19)) OR TS=(SARS-CoV-2)) OR TS=(SARS CoV 2)) OR TS=(2019 Novel Coronavirus Disease)) OR TS=(2019 Novel Coronavirus Infection)) OR TS=(2019-nCoV Disease)) OR TS=(2019 nCoV Disease)) OR TS=(2019-nCoV Diseases)) OR TS=(2019-nCoV)) OR TS=(Coronavirus Disease 2019)) OR TS=(Coronavirus Disease-19)) OR TS=(Coronavirus Disease 19)) OR TS=(Severe Acute Respiratory Syndrome Coronavirus 2 Infection)) OR TS=(SARS Coronavirus 2 Infection)) OR TS=(2019-nCoV Infection)) OR TS=(2019 nCoV Infection)) OR TS=(2019-nCoV Infections)

#3= #1 AND #2

EMBASE

#1'coronavirus disease 2019'/exp OR 'covid-19' OR 'covid 19' OR 'covid19' OR 'sars-cov-2' OR 'sars cov 2' OR '2019 novel coronavirus disease' OR '2019 novel coronavirus infection' OR '2019-ncov disease' OR '2019 ncov disease' OR '2019-ncov diseases' OR '2019-ncov' OR 'coronavirus disease 2019' OR 'coronavirus disease-19' OR 'coronavirus disease 19' OR 'severe acute respiratory syndrome coronavirus 2 infection' OR 'sars coronavirus 2 infection' OR '2019-ncov infection' OR '2019 ncov infection' OR '2019-ncov infections'

#2 'bariatric surgery'/exp OR gastroplast* OR ‘gastric bypass’ OR ‘Roux-en-Y’ OR ‘gastric band’ OR ‘biliopancreatic diversion’ OR gastrectom* OR ‘duodenal switch’ OR ‘gastrointestinal diversion’ OR gastroenterostom* OR ‘jejunoileal bypass’ OR ‘obesity surgery’ OR ‘weight loss

surgery' OR 'weight-loss surgery' OR 'bariatric procedure' OR 'sleeve surgery' OR 'metabolic surgery'

#3= #1AND #2

Cochrane

#1 MeSH descriptor:[Bariatric Surgery] explode all trees OR bariatric surgery OR gastroplast* OR "gastric bypass" OR "Roux-en-Y" OR "gastric band" OR "biliopancreatic diversion" OR gastrectom* OR "duodenal switch" OR "gastrointestinal diversion" OR gastroenterostom* OR "jejunoileal bypass" OR "obesity surgery" OR "weight loss surgery" OR "weight-loss surgery" OR "bariatric procedure" OR "sleeve surgery" OR "metabolic surgery" 16878

#2 MeSH descriptor:[COVID-19] explode all trees OR "COVID-19" OR "COVID 19" OR "COVID19" OR "SARS-CoV-2" OR "SARS CoV 2" OR "2019 Novel Coronavirus Disease" OR "2019 Novel Coronavirus Infection" OR "2019 nCoV Disease" OR "2019 nCoV" OR "Coronavirus Disease 2019" OR "Coronavirus Disease-19" OR "Coronavirus Disease 19" OR "Severe Acute Respiratory Syndrome Coronavirus 2 Infection" OR "SARS Coronavirus 2 Infection" OR "2019 nCoV Infection" OR "2019 nCoV Infections"

#3= #1 AND #2
